# Observing deep radiomics for the classification of glioma grades

**DOI:** 10.1038/s41598-021-90555-2

**Published:** 2021-05-25

**Authors:** Kazuma Kobayashi, Mototaka Miyake, Masamichi Takahashi, Ryuji Hamamoto

**Affiliations:** 1 Division of Medical AI Research and Development, National Cancer Center Research Institute, 5-1-1 Tsukiji, Chuo-ku, Tokyo, 104-0045 Japan; 2 Cancer Translational Research Team, RIKEN Center for Advanced Intelligence Project, 1-4-1 Nihonbashi, Chuo-ku, Tokyo, Japan; 3 Department of Diagnostic Radiology, National Cancer Center Hospital, 5-1-1 Tsukiji, Chuo-ku, Tokyo, 104-0045 Japan; 4 Department of Neurosurgery and Neuro-Oncology, National Cancer Center Hospital, 5-1-1 Tsukiji, Chuo-ku, Tokyo, 104-0045 Japan

**Keywords:** Image processing, Translational research, Magnetic resonance imaging, CNS cancer

## Abstract

Deep learning is a promising method for medical image analysis because it can automatically acquire meaningful representations from raw data. However, a technical challenge lies in the difficulty of determining which types of internal representation are associated with a specific task, because feature vectors can vary dynamically according to individual inputs. Here, based on the magnetic resonance imaging (MRI) of gliomas, we propose a novel method to extract a shareable set of feature vectors that encode various parts in tumor imaging phenotypes. By applying vector quantization to latent representations, features extracted by an encoder are replaced with a fixed set of feature vectors. Hence, the set of feature vectors can be used in downstream tasks as imaging markers, which we call deep radiomics. Using deep radiomics, a classifier is established using logistic regression to predict the glioma grade with 90% accuracy. We also devise an algorithm to visualize the image region encoded by each feature vector, and demonstrate that the classification model preferentially relies on feature vectors associated with the presence or absence of contrast enhancement in tumor regions. Our proposal provides a data-driven approach to enhance the understanding of the imaging appearance of gliomas.

## Introduction

The scientific community has become interested not only in harnessing the predictive performance of machine learning models, but also in dissecting such models to distill useful knowledge that can potentially advance scientific understanding^1^. When a model achieves high prediction performance in a particular task, it is expected to have acquired an expressive internal representation that approximates the explanatory patterns underlying the phenomena of interest. Therefore, the internal representations of trained models can be interpreted to obtain meaningful insights and scientific knowledge without directly observing the phenomena. Based on this concept of acquiring medical knowledge in a data-driven manner, the objective of this study is to discover common features in medical imaging associated with specific clinical information across a patient population.

Particularly, this study focuses on the imaging phenotypes of gliomas, which are the most common central nervous system tumors^2,3^. According to the grading system of the World Health Organization (WHO), gliomas are classified into grades I to IV, based on histopathological findings obtained from surgical biopsies or specimens^4^. Because the degrees of aggressiveness and infiltrative characteristics significantly affect the disease prognosis, the differential diagnosis between lower-grade gliomas (LGG, WHO grades II and III) and high-grade gliomas (HGG, WHO grade IV) is an important issue regarding treatment options and prognosis^5^.

Currently, the standard procedure for classifying tumors according to the WHO grades is based on pathological study. However, there are still many limitations to tumor classification, including the requirement for invasive procedures such as surgical resections or biopsies, inherent sampling errors caused by the heterogeneity of tumors, and the time-consuming process of histopathological analysis. There are also cases wherein it may be dangerous to perform surgical procedures on tumors located at critical sites in the brain. To address these issues, the computational analysis of magnetic resonance imaging (MRI) for tumor grading has attracted significant attention^6,7^. Because MRI can non-invasively observe an entire tumor in vivo, it is free from sampling errors. Therefore, the management of gliomas based on multi-parametric MRI analysis can play a complementary role in pathology-based diagnosis.

Radiomics and deep learning are two mainstays for computational analysis of tumor images. Many intensive studies have attempted to analyze the imaging phenotypes of glioma, and each of these approaches has certain advantages and disadvantages in gaining meaningful insights from trained models.

Radiomics is a research field focusing on decoding tumor phenotypes based on quantitative imaging features^8^. Typically, suitable sets of handcrafted imaging features are extracted from the region of interest (ROI) for analysis. Subsequently, a prediction model based on a machine learning algorithm is trained for a particular prediction task relevant to clinical decision-making. For glioma grading, many previous studies have demonstrated that the tumor characteristics can be quantified using radiomics, and have reported satisfactory discriminative performance^9–12^. Because the radiomics approach uses pre-defined handcrafted imaging features, it has the advantage of high interpretability for the features contributing to the prediction. However, to implement problem-specific handcrafted features, domain knowledge is often required. Because the optimal representative features for a given task are not always obvious, a data-driven approach should be considered to represent the data distribution.

Deep learning has emerged as an innovative technology that enables end-to-end learning between the input data and ground-truth labels^13^. Using backpropagation to tune the parameters of multilayered nonlinear operations during the training process, deep neural networks can automatically abstract useful representations from data. In other words, deep neural networks are capable of data-driven feature extraction. Therefore, a deep learning model can learn internal representations that are meaningful for distinguishing the attributes of samples without relying on feature engineering based on domain knowledge. For example, deep-learning-based algorithms have achieved remarkable prediction performance in glioma grade classification^14–16^. Conversely, in such complex models, a tradeoff between accuracy and explainability has traditionally existed^17^. Hence, complex models, such as deep learning models, are occasionally referred to as black-box models^18^, implying that there is a difficulty in interpreting how the models arrive at a particular outcome.

At the core of our challenge is the internal variability of convolutional neural networks (CNNs). When a CNN is trained to predict the imaging characteristics of gliomas, internal representations can be acquired as low-dimensional feature vectors, which collectively constitute the feature maps. One may argue that these feature vectors can then be used as imaging markers in downstream tasks because they are expected to adequately represent the appearance of tumors. Nevertheless, only a few studies have deeply investigated different types of imaging characteristics exploited by deep learning models for prediction in clinical tasks of glioma imaging. Among existing studies, Banerjee et al.^15^ investigated the properties of convolutional kernels in different layers through visualization. However, the internal variability of the typical CNNs still hinders model interpretability, whereby each feature map changes dynamically depending on individual inputs, especially focusing on determining the types of internal representations that are critical for a specific task. Because the objective of the majority of medical studies is to find specific factors that are significantly common in a diseased population, it is crucial to fix the variability of feature vectors representing targeted imaging phenotypes.

To combine the advantages of radiomics and deep learning by solving the internal variability of CNNs, we propose a straightforward approach to incorporate vector quantization into the feature extraction process of deep learning models. Particularly, we apply vector quantization to the latent representation inside a segmentation model based on an encoder–decoder structure for tumor regions in images. Through the process of vector quantization, individually varying features extracted from an encoder can be replaced with a fixed set of feature vectors, the configuration of which is also optimized in the model training process. As a result, each imaging phenotype can be indicated by a shareable set of feature vectors, allowing themselves to be used as imaging markers for downstream tasks. Subsequently, we attempt to identify specific types of internal representations associated with particular clinical information by training a classification model based on the set of feature vectors. Thus, our approach combines the flexible representative capacities of deep learning and the highly interpretable aspects of radiomics to acquire meaningful knowledge in a data-driven manner, which we call *deep radiomics*. Additionally, we devise a feature ablation study to visualize which types of imaging characteristics are utilized by the classification model to provide interpretable feedback to physicians for the task-specific radiological findings. We also discuss whether the obtained result is consistent with the findings reported in the literature.

## Methods

In this section, we describe a method to extract a shareable set of feature vectors inside a segmentation network by incorporating vector quantization and to utilize them for the classification of glioma grades using logistic regression. The latter task was formulated as a binary classification whereby an input magnetic resonance (MR) volume is diagnosed either as LGG or HGG. Additionally, the types of imaging characteristics that enable the prediction were investigated by conducting a feature ablation study.

### Dataset

We prepared a dataset of brain MRIs with gliomas from the 2019 BraTS Challenge^19–22^. This dataset contains T1, Gd-enhanced T1, T2, and FLAIR sequences for patients diagnosed with LGG or HGG. Note that LGG stands for “lower-grade” glioma herein, the definition of which includes both low-grade glioma (WHO grade II) and intermediate-grade glioma (WHO grade III)^5,23^. Bakas et al.^24^ gives the detailed description of scanning and annotation protocols. Briefly, all clinically acquired multi-parametric MRI scans were co-registered to a common anatomical template, resampled to $1 \;{\mathrm {mm}}^3$, and underwent skull-stripping.

In this study, all four sequences were used, and three types of datasets were obtained: a training dataset (MICCAI_BraTS_Training) containing 355 patients, a validation dataset (MICCAI_BraTS_Validation) containing 125 patients, and a test dataset (MICCAI_BraTS_Testing) containing 167 patients. Only MICCAI_BraTS_Training contains a patient-basis diagnosis of LGG (76 patients) and HGG (259 patients) that is pathologically confirmed^24^. MICCAI_BraTS_Training originally contained three ground-truth segmentation labels for abnormalities: Gd-enhanced tumor (ET), peritumoral edema (ED), and necrotic and non-enhancing tumor core (NET). Under the supervision of expert radiologists, we segmented the images in MICCAI_BraTS_Validation and MICCAI_BraTS_Testing into the aforementioned three abnormal categories (ET, ED, and NET). Note that the names of the datasets given in the 2019 BraTS Challenge and the purpose of using each dataset in this study are different. To train a segmentation network, a dataset obtained by concatenating MICCAI_BraTS_Validation and MICCAI_BraTS_Testing was used as a *training dataset*. After training the segmentation network, a classification model was constructed based on MICCAI_BraTS_Training as a *validation dataset*, which is the only dataset containing information on the glioma grades.

### Proposed algorithm for deep radiomics

Here, we describe the algorithm for extracting and exploiting deep radiomics for the classification of glioma grades.

#### Overview of the algorithm

**Figure 1:**
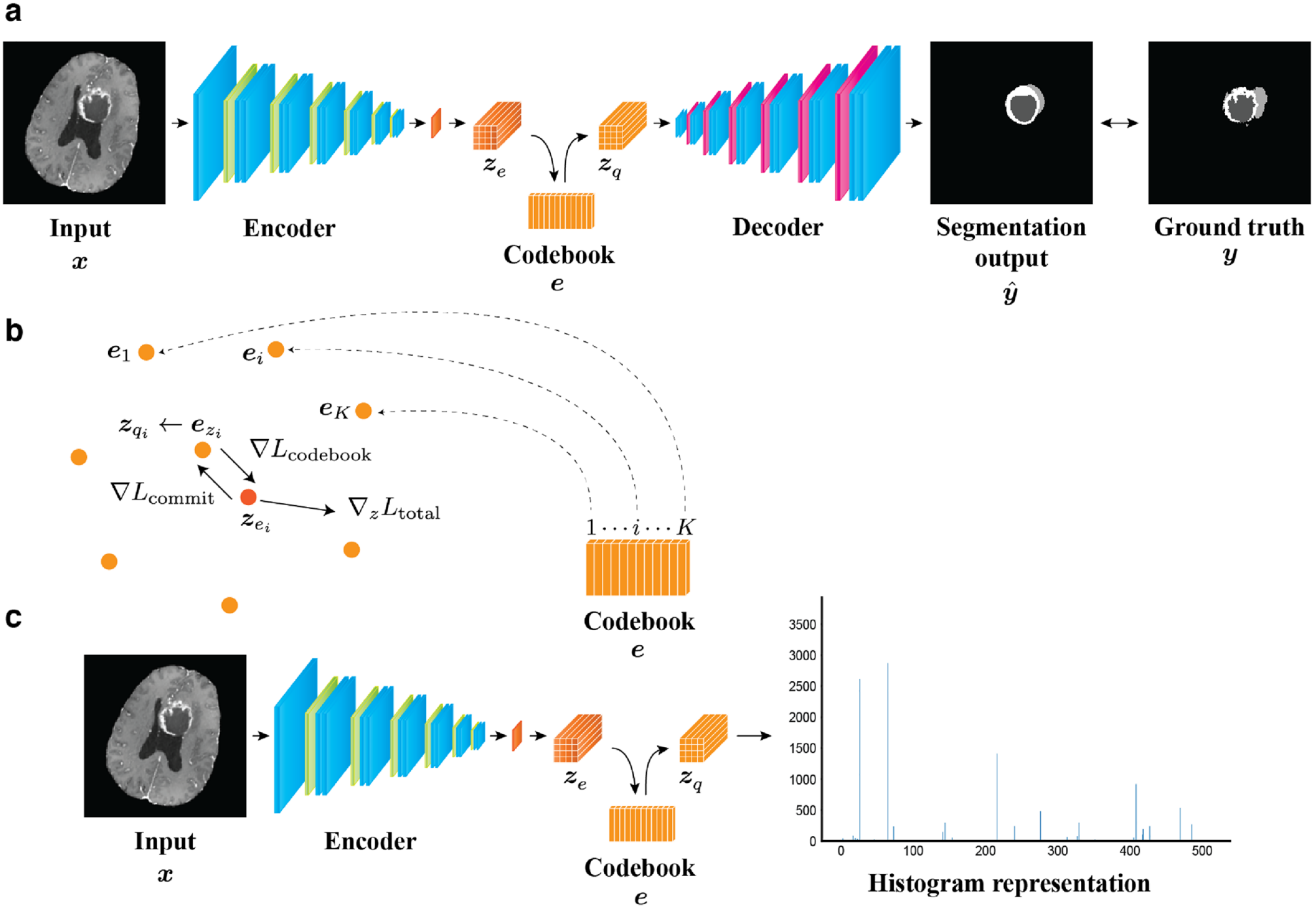
Obtaining a shareable set of feature vectors from a segmentation network. (**a**) A segmentation network consists of an encoder–decoder pair and stores a shareable set of feature vectors in a codebook. At the training stage of a tumor segmentation pre-task, an input image $\varvec{x}$ is mapped onto a latent representation $\varvec{z}_e$ through the encoder. Vector quantization is performed based on the codebook $\varvec{e}$ by replacing each feature vector in $\varvec{z}_e$ with the nearest codeword to produce a quantized latent representation $\varvec{z}_q$. Then, the decoder produces a segmentation output by taking $\varvec{z}_q$ as the input. The error between the segmentation output and a ground-truth label is evaluated to train the network. (**b**) During the training, the codebook loss $\nabla L_{\mathrm {codebook}}$ enforces the codebook variables toward the encoder’s output, meanwhile the commitment loss $\nabla L_{\mathrm {commit}}$ exerts the opposite effect. To alter the configuration of the codebook, the encoder’s output is updated for the next forward pass according to the learning objective $\nabla _z L_{\mathrm {total}}$. (**c**) When using the shareable set of feature vectors in a downstream task, the encoder is employed as a feature extractor. The latent representation of an input image is mapped onto the quantized latent representation $\varvec{z}_q$, and then a histogram representation is constructed. This histogram representation contains information on the frequency with which each feature vector appears in the input image.

**Figure 2:**
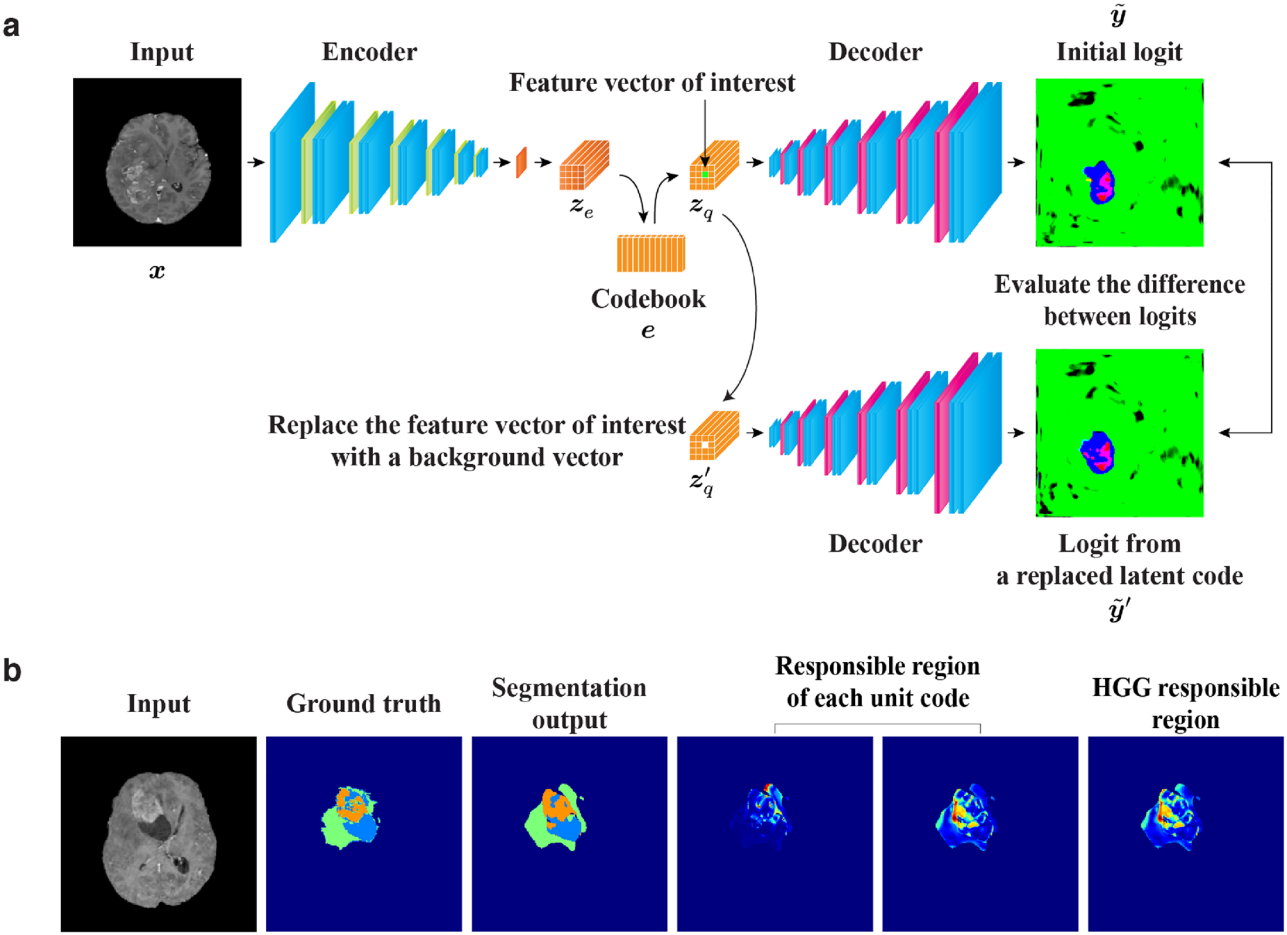
Overview of feature ablation study conducted to visualize the image region encoded by each feature vector. (**a**) The input image is initially mapped onto the quantized latent representation $\varvec{z}_q$ through the encoder, which functions as a feature extractor. This initial latent representation is subsequently fed into the decoder to generate the segmentation output $\hat{\varvec{y}}$, and the logit map $\tilde{\varvec{y}}$ obtained before the final argmax operation is retained in the subsequent procedure. Then, the feature vector of interest in $\varvec{z}_q$ is replaced with a background vector to generate the replaced latent representation $\varvec{z}_q^\prime$. The background vector is identified as the most common feature vector in the background of the images (that is, the region outside the body). Next, the decoder outputs the logit map $\tilde{\varvec{y}}^\prime$ again by taking $\varvec{z}_q^\prime$ as the input. Because the difference between $\tilde{\varvec{y}}$ and $\tilde{\varvec{y}}^\prime$ reflects the image region affected by the replacement, the difference map is referred to as the *responsible region* of the feature vector of interest. (**b**) The two responsible regions corresponding to the HGG responsible vectors are shown along with examples of an input image, ground-truth label, and segmentation output. By collecting the responsible regions from all responsible vectors for a particular glioma grade, we can observe the relation between the type of imaging characteristics and glioma grade.

We first train an encoder–decoder network to predict the segmentation of glioma imaging characteristics from a two-dimensional (2D) axial slice of multi-parametric MRI (Fig. 1a). The core of our proposal is to perform vector quantization at the bottom of the segmentation network, where a codebook consisting of a fixed number of feature vectors as codewords is trained to capture the imaging characteristics meaningful for the tumor segmentation (Fig. 1b). After the training, for individual input images, the varying feature representations by the encoder are substituted by the codewords located at fixed positions through vector quantization. The codewords in the learned codebook can be regarded as *shareable* in the dataset. Subsequently, imaging features of each MRI volume are represented as a histogram, which summarizes how many times each codeword in the codebook appears in each slice of the MRI volume (Fig. 1c). Thereafter, by applying simple logistic regression to classify different glioma grades based on the histogram representation, a set of feature vectors that are significantly associated with the prediction is identified. We further conduct a feature ablation study to visualize which types of imaging characteristics are associated with glioma grades in the image space (Fig. 2).

#### Notation

Let us consider a multi-parametric three-dimensional (3D) MRI volume $\varvec{X} \in {\mathbb {R}}^{C \times W \times H \times I}$, where *C* is the number of channels, *W* and *H* represent the height and width of the axial slices, respectively, and *I* is the number of axial slices. We define $\varvec{x} \in {\mathbb {R}}^{C \times W \times H}$ as a slice in the axial view. The segmentation network encodes a slice-wise input $\varvec{x}$ into the low-dimensional latent representation $\varvec{z} \in {\mathbb {R}}^{C^{\prime } \times W^{\prime } \times H^{\prime }}$ and decodes the segmentation output $\hat{\varvec{y}} \in {\mathbb {R}}^{S \times W \times H}$, where *S* is the number of segmentation labels. The ground-truth segmentation label $\varvec{y} \in {\mathbb {R}}^{S \times W \times H}$ is used to train the segmentation network. The series of latent representations $\varvec{z}$ for each slice of the MRI volume can be concatenated into a summarized representation $\varvec{Z} \in {\mathbb {R}}^{C^{\prime } \times W^{\prime } \times H^{\prime } \times I^{\prime }}$, which is considered as a volume-based representation. The glioma grades are classified on a volume basis because grading is carried out for each patient based on pathological examinations^24^.

#### Segmentation networks with a shareable set of feature vectors

A segmentation network was trained to extract a shareable set of feature vectors. As shown in Fig. 1a, the network consisted of an encoder–decoder pair connected via a discrete latent space containing a set of feature vectors as codewords in a codebook. Through the encoder, a 2D MRI slice $\varvec{x}$ is mapped to a latent representation $\varvec{z}_e$, which can be variable according to individual inputs. In the latent space, vector quantization is performed based on a codebook $\varvec{e} = \{e_k |k = 1, \ldots , K\}\in {\mathbb {R}}^{K \times D}$, which stores a shareable set of *K* feature vectors as codewords $e_k \in {\mathbb {R}}^D$, by replacing each feature vector in $\varvec{z}_e$ with the nearest codeword to produce the quantized latent representation $\varvec{z}_q$. This vector quantization process is analogous to that of a vector-quantized variational autoencoder (VQ-VAE)^25,26^. As illustrated in Fig. 1b, the feature vectors corresponding to each voxel of $\varvec{z}_e$ are quantized by executing a nearest-neighbor lookup on the codebook, as follows:

1 $$\begin{aligned} z_i = {\mathop {\mathrm{arg\, min}}\limits _{k \in [K]}} \Vert \varvec{z}_{e_i} - e_k\Vert _2. \end{aligned}$$

Thereafter, the codewords in the codebook are collected as a quantized latent representation $\varvec{z}_q$, as follows:

2 $$\begin{aligned} \varvec{z}_{q_i} = e_{z_i}. \end{aligned}$$

To optimize this process, the codebook and encoder are trained to minimize the objective, which is referred to as *latent loss*, as follows:

3 $$\begin{aligned} L_{{\mathrm {latent}}} = \Vert {\mathrm {sg}}[\varvec{z}_e(x)] - \varvec{e}\Vert ^2_2 + \beta \Vert \varvec{z}_e(x) - {\mathrm {sg}}[\varvec{e}]\Vert ^2_2, \end{aligned}$$

where ${\mathrm {sg}}$ represents the stop-gradient operator; this serves as an identity function at the forward computation time and has zero partial derivatives. During training, the *codebook loss*, which is the first term in the aforementioned equation, updates the codebook variables by delivering the codewords to the encoder’s output (see the arrow indicated by $\nabla L_{\mathrm {codebook}}$ in Fig. 1b). Simultaneously, the *commitment loss*, which is the second term, encourages the output of the encoder to move closer to the target codewords (see the arrow indicated by $\nabla L_{\mathrm {commit}}$ in Fig. 1b). The hyperparameter $\beta$ controls the reluctance of changing the encoder output to match the corresponding codewords. Backpropagation or exponential moving average can be used to train the codebook^27^. Notably, the size of the codebook can be arbitrarily tuned, which ensures that a certain amount of information is preserved and compressed within the latent space^26^.

Then, the decoder takes $\varvec{z}_q$ as input and generates the segmentation map $\hat{\varvec{y}}$, which is encouraged to be similar to the ground-truth labels $\varvec{y}$. The segmentation loss function consists of the soft Dice^28^ and focal losses^29^. In summary, the overall training objectives for the segmentation network are as follows:

4 $$\begin{aligned} L_{\mathrm {total}} = L_{\mathrm {latent}} + L_{\mathrm {segmentation}}. \end{aligned}$$

At each iteration to minimize Eq. (4), the encoder output $\varvec{z}_e$ is updated to alter the configuration in the next forward pass (see the arrow indicated by $\nabla _z L_{\mathrm {total}}$ in Fig. 1b). Consequently, after the training of the tumor segmentation, we can consider the codewords as a shareable set of feature vectors that contain the representations describing imaging phenotypes of gliomas. Hereinafter, the image analysis method exploiting this shareable set of feature vectors obtained in a data-driven manner is called deep radiomics.

#### Histogram representation of brain MRI based on deep radiomics

We hypothesize that these feature vectors can be useful to distinguish between LGG and HGG. To demonstrate this, we start with a volume-wise representation of brain MRI, as the pathologically-confirmed glioma grade is associated with the entire volume. We build upon the encoder followed by the vector quantization used as a feature extractor *f* to produce the slice-wise quantized latent representation $\varvec{z}_q$ (Fig. 1c). All *I* quantized latent representations $\{\varvec{z}_1, \ldots , \varvec{z}_I\}$ extracted from slices $\{\varvec{x}_1, \ldots ,\varvec{x}_I\}$ in the MRI volume $\varvec{X} \in {\mathbb {R}}^{C \times W \times H \times I}$ are concatenated into a volume-wise representation $\varvec{Z}_q$. Subsequently, we convert this representation into a histogram representation to approximate the imaging appearance as a count of each feature vector on a volume basis, as follows:

5 $$\begin{aligned} \varvec{Z}_q = \sum _{i \in I} f(\varvec{x}) = \sum _{i \in I} \varvec{z}_q \approx \sum _{i \in I} H_{k \in K} (c_{k_i}, e_k) = H_{k \in K} (c_k, e_k), \end{aligned}$$

where *H* is an operator to rearrange a histogram according to the number of feature vectors, *K* is the number of discrete feature vectors in the codebook, $c_{k_i}$ is the number of occurrence of the $k\hbox {th}$ feature vector in the $i\hbox {th}$ axial slice, and $c_k$ is the summed occurrence of the $k\hbox {th}$ feature vector appearing in the MRI volume.

#### Classification models for glioma grades

A key benefit of the vector quantization is that a specific set of feature vectors stored in the codebook can be shared across a population, fixing the variability of internal representations of CNNs. This allows us to use these feature vectors as imaging markers for downstream tasks. Therefore, to establish a binary classification model to discriminate the glioma grade, we used logistic regression based on the histogram representation. By considering the number of occurrences $c_i$ of each feature vector as an explanatory variable, the logistic regression model can be formulated as follows:

6 $$\begin{aligned} {\mathrm {logit}} (p) = \beta _0 + \sum _{k \in K^{*}} \beta _k c_k, \end{aligned}$$

where *p* indicates the probability of a particular class, $\beta$ is a regression coefficient, and $K^{*}$ denotes a set of significant classifier coefficients based on the effect likelihood ratio test. The classification performance was evaluated based on accuracy, precision, recall (sensitivity), specificity, and negative predictive value, where HGG and LGG were considered as positive and negative, respectively.

#### Robustness assessment of the deep radiomics

Robustness of features under varying scanning and segmentation conditions is a significant challenge in conventional radiomics^30^. Several researchers have studied reproducibility of radiomics and report the variability of radiomics features depending on image preprocessing techniques such as voxel size, slice thickness, and normalization methods^31–33^. Therefore, robustness assessment of the deep radiomics is necessary to demonstrate its usefulness in medical image analysis.

We evaluated the robustness of the deep radiomics from two perspectives. First, we investigated the reproducibility of the volume-wise representation as the histogram shown in Eq. (5). As standardization of pixel/voxel intensity in brain MRIs significantly affects radiomics markers^31,34^, we imposed perturbations by scaling and shifting the entire pixel value of input images. Then, the extent to which selected feature vectors deviated from the original histogram, which was acquired without any perturbation, was quantified. This is formulated as an index called *difference ratio* as follows:

7 $$\begin{aligned} \text{ difference } \text{ ratio } = \frac{\text{ number } \text{ of } \text{ feature } \text{ vectors } \text{ different } \text{ from } \text{ the } \text{ original } \text{ histogram }}{\text{ number } \text{ of } \text{ feature } \text{ vectors } \text{ in } \text{ the } \text{ original } \text{ histogram }}, \end{aligned}$$

where the numerator is calculated as the sum of the absolute values of the difference in the number of occurrences of each feature vector. Second, we assessed the performance degradation of the classification model in Eq. (6) under the same perturbations. The performance indices, such as accuracy, precision, recall (sensitivity), specificity, and negative predictive value, were calculated according to the magnitude of the perturbations.

#### Identification of responsible vectors

For interpretability, linear models such as logistic regression are considered as *transparent*, whereas complex models involving deep learning are sometimes regarded as *black-box*^35^. Transparent models are considered so because they are inherently interpretable. For example, statistical tests of individual predictors for a logistic regression model showing goodness of fit for a target observation can identify significant variables for prediction. Therefore, we sought to identify feature vectors with coefficients that exhibited statistical significance using the effect likelihood ratio test, which is indicated by $K^*$ in Eq. (6). We refer to these significant feature vectors as *responsible vectors*. Then, to elucidate the preference of each responsible vector for either LGG or HGG, we analyzed the frequency of each responsible vector according to the glioma grade using the Wilcoxon signed-rank test, because the null-hypothesis for the normality of the variable distribution was rejected by the Shapiro–Wilk test. If a responsible vector is significantly frequent in LGG patients, it is called an *LGG responsible vector*. Similarly, *HGG responsible vectors* are defined as frequent feature vectors in HGG patients. The level of statistical significance was set to $p < 0.05$.

#### Feature ablation study to visualize responsible regions

To enhance the interpretability of deep radiomics, we further devise a feature ablation study to visualize the imaging characteristics that are encoded by a specific feature vector (Fig. 2). First, an input image is projected onto a corresponding latent representation by the encoder and the vector quantization (Fig. 2a). The quantized latent representation $\varvec{z}_q$ is then fed into the decoder to generate the logit map $\tilde{\varvec{y}}$, which is subsequently converted into the segmentation output $\hat{\varvec{y}}$ through the argmax function. Here, the logit map $\tilde{\varvec{y}}$ is retained for further processing. Next, the feature vector of interest in the initial latent representation $\varvec{z}_q$ is replaced with a background vector, which is defined as the most common vector in the background of the images (that is, the black region outside the body in MRI). The replaced latent representation $\varvec{z}_q^\prime$ is subsequently input into the decoder and the corresponding logit map $\tilde{\varvec{y}}^\prime$ is retained. Finally, the per-pixel L1 difference between the two logit maps, $\tilde{\varvec{y}}$ and $\tilde{\varvec{y}}^\prime$, is evaluated. Because the difference map reflects the changed segmentation output through the removal of the feature vector of interest, we can assess the imaging characteristics encoded by each feature vector by observing the corresponding region in the input image. Therefore, we call this difference map the *responsible region* (Fig. 2b). The responsible regions from all LGG responsible and HGG responsible vectors are collectively denoted as the *LGG responsible region* and *HGG responsible region*, respectively.

For a quantitative assessment, the values of the responsible region (the per-pixel L1 difference between $\tilde{\varvec{y}}$ and $\tilde{\varvec{y}}^\prime$) was calculated according to each segmentation label (ET, ED, and NET). The null-hypotheses for the normality of these values in the LGG and HGG responsible regions were rejected by the Shapiro–Wilk test ($p < 0.05$). Thus, we performed the Kruskal–Wallis test and the non-parametric comparisons for all pairs (NET-ED, ED-ET, and NET-ET) using the Dunn method for joint ranking to reveal the responsible regions that are significantly associated with a particular tumor region.

### Implementation details

The segmentation network was implemented and trained according to the following descriptions.

#### Preprocessing

All four sequences, T1, Gd-enhanced T1, T2, and FLAIR, were concatenated into a four-channel MR volume $\varvec{X} \in {\mathbb {R}}^{4 \times 240 \times 240 \times 155}$. The preprocessing pipeline, including axial image resizing to $256 \times 256$ and Z-score normalization, was performed. Moreover, each three-dimensional (3D) MR volume was decomposed into a collection of 2D axial slices $\{\varvec{x}_1, \varvec{x}_2, \dots , \varvec{x}_{155} \in {\mathbb {R}}^{4 \times 256 \times 256} \}$. Both the training and validation datasets were preprocessed.

#### Encoder network

The encoder consists of residual blocks^36^, wherein two [*convolution* + *group normalization*^37^ + *LeakyReLU*] sequences are processed with residual connection. The kernel size, stride, and padding size of the convolution function in the residual blocks are set to 3, 1, and 1, respectively. From the first to the last residual blocks, the encoder uses $32 - 64 - 128 - 128 - 128 - 128$ filter kernels. Each residual block is followed by a downsampling block to halve the feature map size, except for the bottom of the network. The downsampling block consists of a sequence of [*convolution* + *group normalization* + *LeakyReLU*], whose kernel size, stride, and padding size are set to 3, 2, and 1, respectively. The input image is required to have a size of $4 \times 256 \times 256$ $(= {\mathrm {channel}} \times {\mathrm {height}} \times {\mathrm {width}})$. The encoder output, which is denoted as $\varvec{z}_e$, has a size of $64 \times 8 \times 8$.

#### Decoder network

The decoder architecture is approximately symmetrical to that of the encoder. From the first to the last residual block, the decoder uses $128 - 128 - 128 - 128 - 64 - 32$ filter kernels. The residual blocks consist of two [*convolution* + *group normalization* + *LeakyReLU*] sequences that follow an upsampling layer using an interpolation function coupled with a convolutional function to double the size of the feature map. Latent variables sampled from $p(\varvec{z})$ with a size of $64 \times 8 \times 8$ pass through the decoder to yield reconstructed 2D images with a size of $4 \times 256 \times 256$.

#### Training setups

All neural networks were implemented using Python 3.7 with the PyTorch library 1.6.0^38^ on an NVIDIA Tesla V100 GPU with CUDA 10.0. The initialization method proposed by He et al.^39^ was applied to all the networks. Adam optimization^40^ with a learning rate of $1 \times 10^{-4}$ was used for the segmentation network. The other hyperparameters were empirically determined as follows: batch size = 72, maximum number of epochs = 600. The size of the latent codebook was $512 \times 64$ ($= K \times D$). During training, the data augmentation included horizontal flipping, random rotation, and random-intensity shifting and scaling.

## Results

### Segmentation performance of segmentation network

Comparison of voxel volumes according to each tumor region (ET, ED, and NET) for the two glioma grades is shown in Table 1. The segmentation performance of the segmentation network based on the Dice score (mean ± standard deviation) was as follows: $0.56 \pm 0.28$ for NET, $0.68 \pm 0.16$ for ED, $0.69 \pm 0.23$ for ET, $0.80 \pm 0.19$ for the tumor core (NET $+$ ET), and $0.76 \pm 0.12$ for the whole tumor (NET $+$ ED $+$ ET). These intermediate Dice scores were expected, because the segmentation network has a bottleneck where the imaging features are compressed according to the limited size of the codebook. Notably, the primary objective of the segmentation network is not segmentation, but to provide a shareable set of feature vectors that sufficiently cover the imaging phenotypes of gliomas and are discriminative in downstream tasks.

**Table 1:** Comparison of voxel volumes (mean ± standard deviation) [${\mathrm {cm}}^3$] according to each tumor regions between LGG and HGG in the validation dataset (MICCAI_BraTS_Training).

	LGG	HGG
NET	$52.4 \pm 47.3$	$13.8 \pm 15.9$
ED	$53.7 \pm 48.7$	$58.7 \pm 38.3$
ET	$5.5 \pm 13.5$	$22.6 \pm 18.6$
WT	$111.5 \pm 76.7$	$95.1 \pm 54.8$

NET: necrotic and non-enhancing tumor core, ED: peritumoral edema, ET: Gd-enhanced tumor, WT: whole tumor (= NET + ED + ET).

### Histogram representation

**Figure 3:**
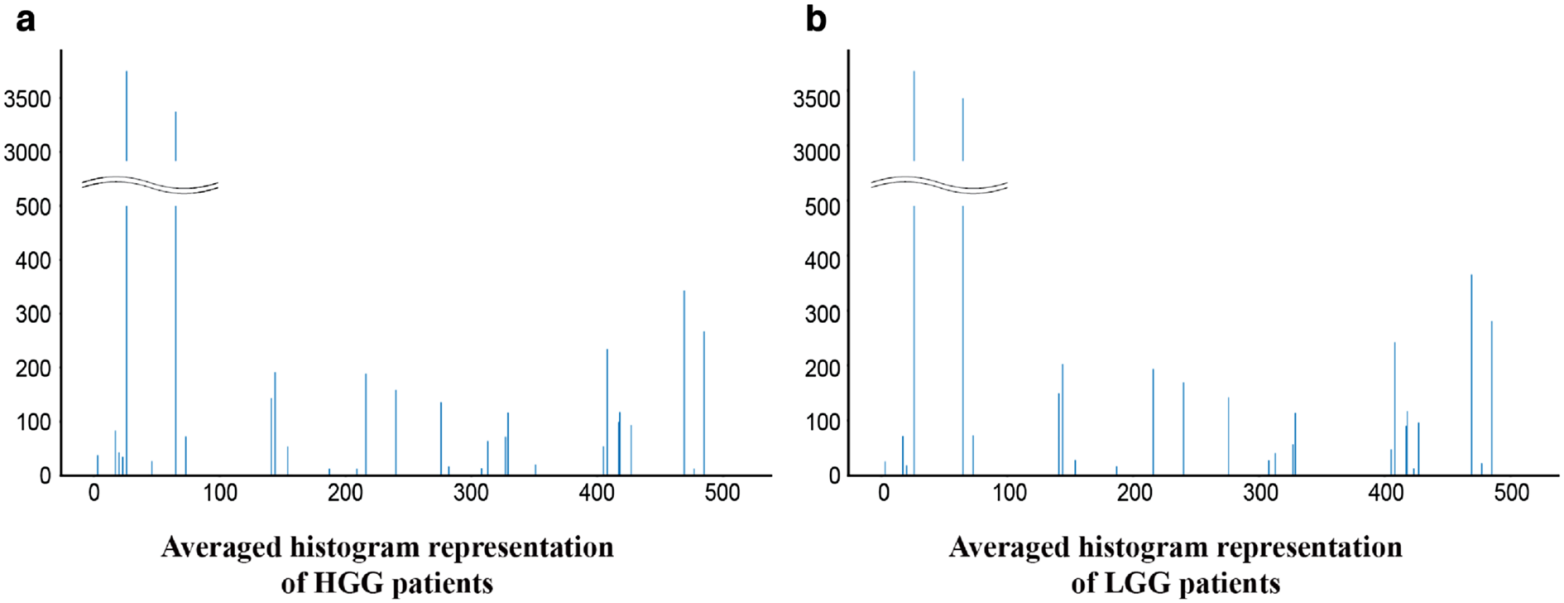
Average histogram representation for patients with (**a**) HGG and (**b**) LGG.

**Figure 4:**
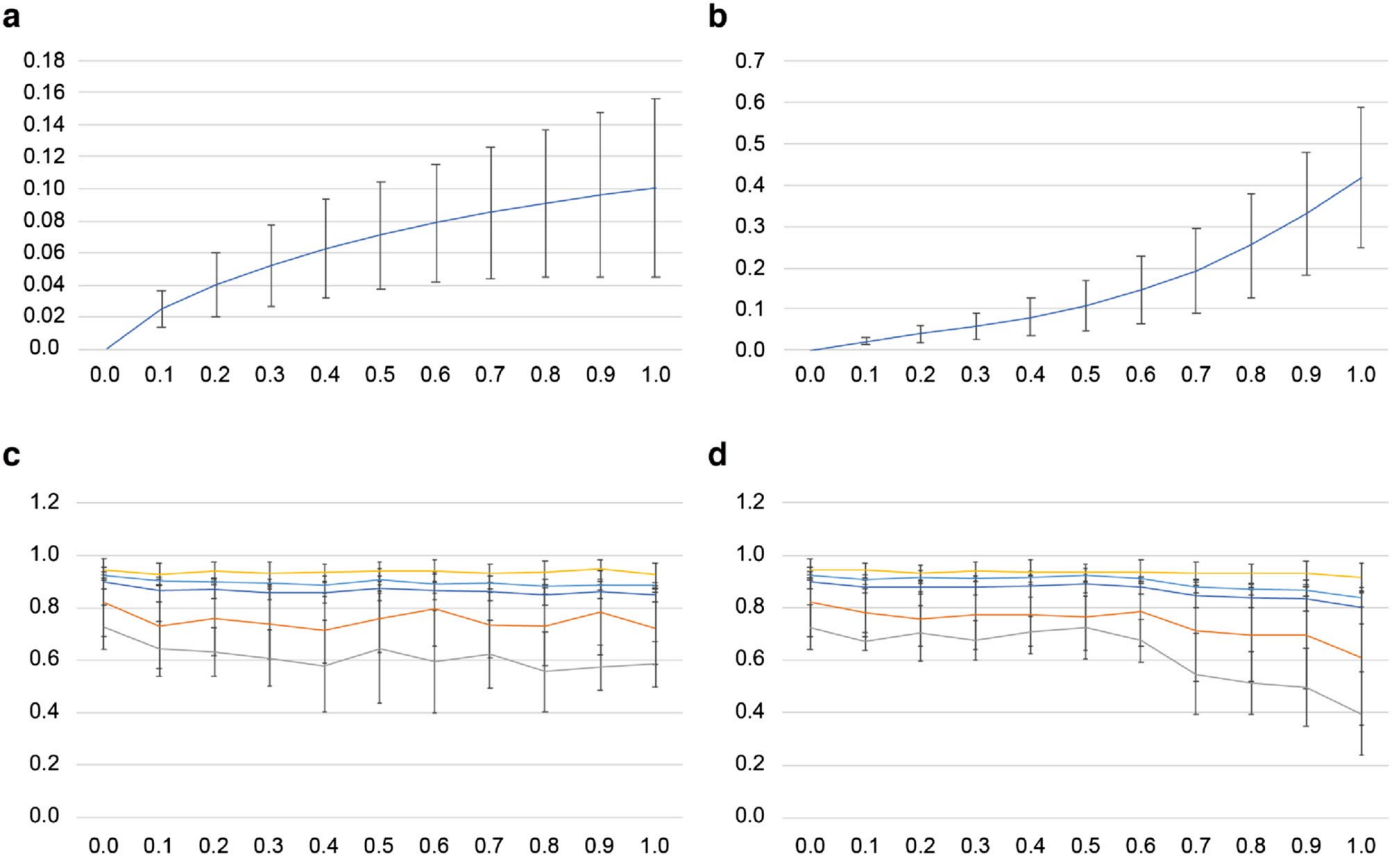
Assessment of the robustness of deep radiomics. Each perturbation such as pixel intensity scale and shift was applied to input images with the magnitudes in the range between 0.0 and 1.0 in increments of 0.1. (**a**) Difference ratio according to pixel intensity scale. (**b**) Difference ratio according to pixel intensity shift. (**c**) Classification performances (accuracy: blue, precision: orange, recall (sensitivity): gray, specificity: yellow, and negative predictive value: light blue) according to pixel intensity scale. (**d**) The same classification performances according to pixel intensity shift. See the performance degradation owing to the pixel intensity shift worsened when the magnitude exceeds more than 0.6. For all the data points, mean ± standard deviation is indicated.

Figure 3 shows the average histogram representations of HGG and LGG patients. These histograms indicate the average number of times each feature vector appears per MRI volume according to the glioma grading. A slight difference can be observed between these two histograms, particularly regarding low-frequency feature vectors. Figure 4a,b shows the difference ratio (Eq. 7), which indicates the reproducibility and repeatability of the same histogram representation under perturbations. For pixel intensities that were standardized through Z-score normalization, we applied scaling (Fig. 4a) and shifting (Fig. 4b) of pixel values with different magnitudes ranging from 0.0 to 1.0 in increments of 0.1 as perturbations. As can be seen, the difference ratio increased as the degree of perturbation increased. Further, shifting tended to have a larger impact than scaling.

### Classification accuracy

According to fivefold cross-validation in the validation dataset, the classification results (mean ± standard deviation) of the glioma-grading model were as follows: $0.90 \pm 0.03$ of accuracy, $0.82 \pm 0.13$ of precision, $0.73 \pm 0.08$ of recall (sensitivity), $0.95 \pm 0.04$ of specificity, and $0.93 \pm 0.01$ of negative predictive value. As for the robustness of the classification model under the same perturbations, shifting (Fig. 4d) tended to entail larger decline in the performance than scaling (Fig. 4c). The performance degradation seemed to be consistent with the degree of difference ratio caused by each level of the perturbations.

### Identification of responsible vectors

After evaluating the classification performance based on fivefold cross-validation, we trained the classification model again on all samples for further analysis. Additionally, the classification model identified two HGG responsible vectors and three LGG responsible vectors, which were significant covariates in the logistic regression models (effect likelihood ratio test: $p < 0.05$) and had significantly uneven distribution according to the glioma grading (Wilcoxon signed-rank test: $p < 0.05$).

### Qualitative evaluation of responsible regions

As demonstrated by the classification performance, the feature vectors in the codebook appear to represent the imaging characteristics of gliomas and may convey meaningful information to identify the glioma grade. Therefore, we investigated the types of imaging characteristics that are encoded by each feature vector through feature ablation study (Fig. 2). We visualized both the HGG and LGG responsible regions to evaluate the overlap with the segmented tumor regions that were provided as ground-truth labels (ET, ED, and NET).

**Figure 5:**
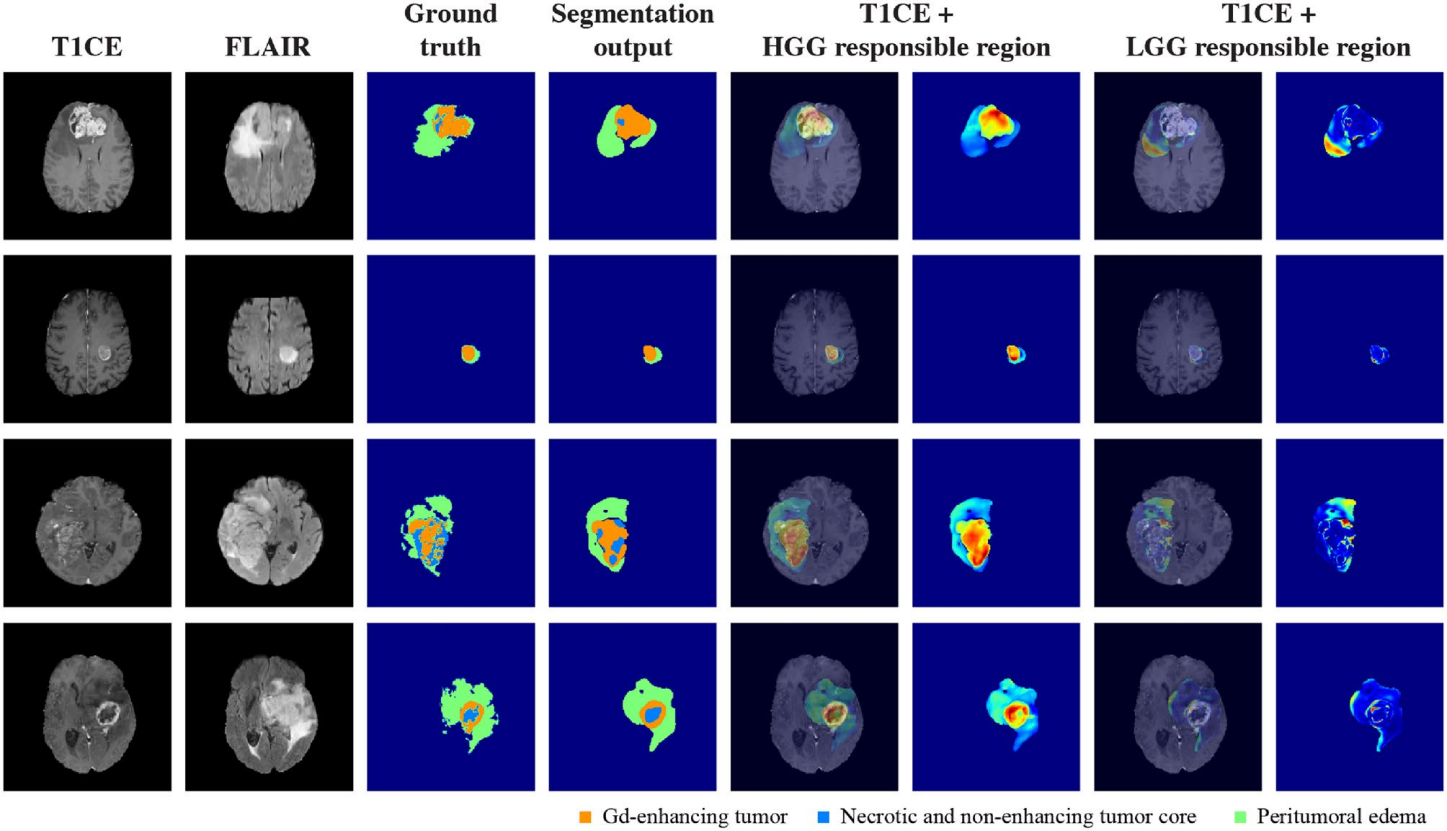
Example results for responsible regions in HGG patients. For patients with HGG, the Gd-enhanced T1 (T1CE) and FLAIR sequences, ground-truth labels, segmentation outputs, HGG responsible regions, and LGG responsible regions are shown. The tumor regions are adequately correlated with the HGG responsible regions, but overlap with the LGG responsible regions is scarce. The color map indicates the high-difference values in red and the lower-difference values in blue; the values are standardized for each patient.

Figure 5 shows the distribution of the HGG and LGG responsible regions in patients with HGG. Notably, the HGG responsible regions were strongly correlated with the tumor regions of the HGG patients. The large difference values (indicated in red color) were preferentially gathered in the central region of the tumor corresponding to the ET label. By contrast, although a small overlap with the LGG responsible regions was observed in the peripheral regions of the tumor, the values were relatively low as indicated by the color map.

**Figure 6:**
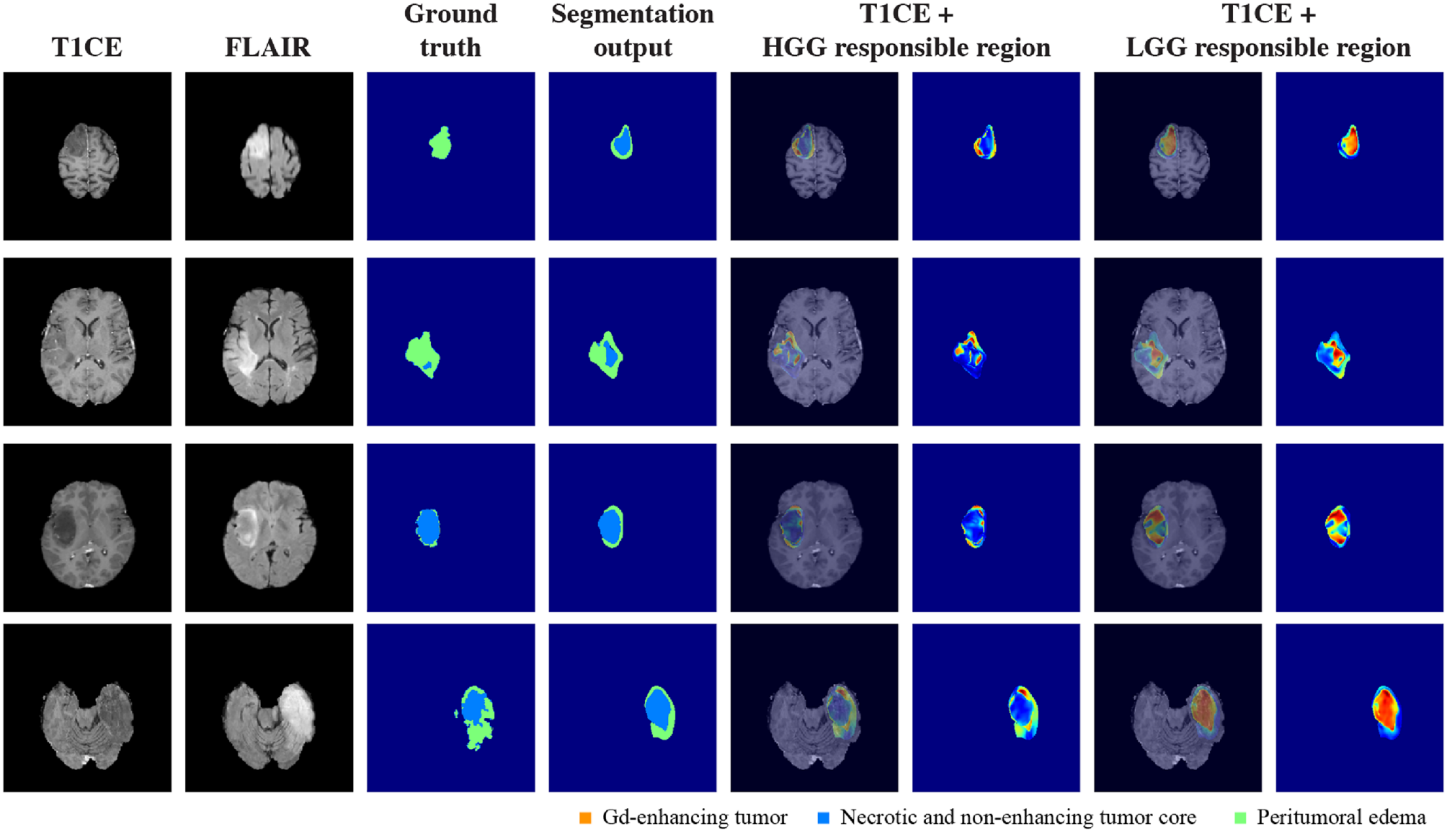
Example results for responsible regions in LGG patients. For patients with LGG, the Gd-enhanced T1 (T1CE) and FLAIR sequences, ground-truth labels, segmentation outputs, HGG responsible regions, and LGG responsible regions are shown. The tumor regions are strongly correlated with the LGG responsible regions, particularly in the central area of the tumor. The overlap with the HGG responsible regions is relatively insignificant and peripherally distributed at best. The color map indicates the high-difference values in red and the low-difference values in blue; the values are standardized for each patient.

Figure 6 presents the distribution of HGG and LGG responsible regions in patients with LGG. In contrast to the aforementioned results, the LGG responsible regions significantly overlapped with the central region of the tumor, and particularly the region labeled as NET. The signals of the HGG responsible regions were not remarkable, as indicated by their low values.

### Quantitative evaluation of responsible regions

**Figure 7:**
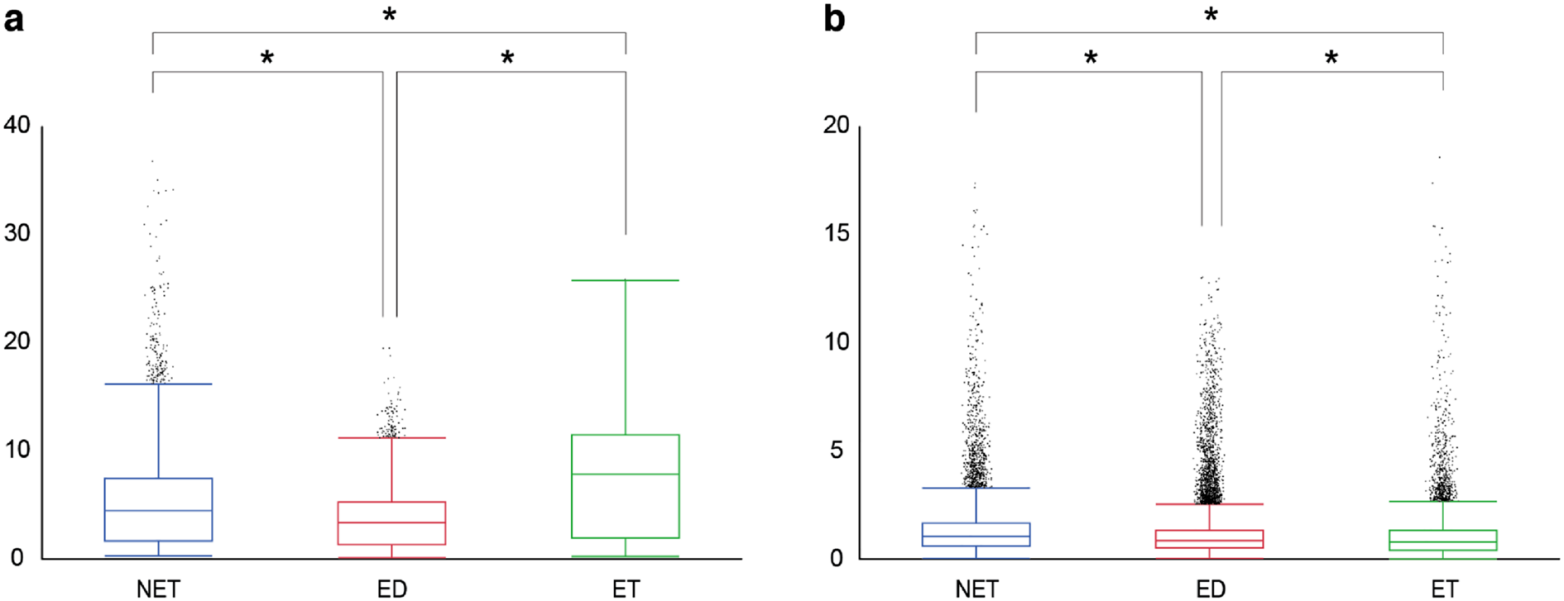
Quantitative evaluation of overlap between responsible regions and segmentation labels. (**a**) Difference values of HGG responsible regions in each segmentation label: Gd-enhanced tumor (ET), peritumoral edema (ED), and necrotic and non-enhancing tumor core (NET). The values in the ET region are the highest among the three segmentation categories. (**b**) Difference values of LGG responsible regions for the same segmentation labels. The NET regions have the highest values; * indicates a statistical significance $< 0.0001$.

Finally, we quantitatively evaluated the preferences of each responsible region according to the ET, ED, and NET segmentation labels. The difference values in each segmented area were summed and statistically compared, as shown in Fig. 7. For the HGG responsible regions, the mean ± standard deviation values for the NET, ED, and ET labels were $5.48 \pm 4.69$, $3.78 \pm 2.79$, and $7.66 \pm 5.37$, respectively. The Kruskal–Wallis test and the non-parametric comparisons carried out for all pairs using the Dunn method for joint ranking revealed that the highest values appeared in the ET region ($p < 0.0001$). For the LGG responsible regions, the values for the NET, ED, and ET labels were $1.22 \pm 1.26$, $1.02 \pm 1.10$, and $0.92 \pm 1.02$, respectively. The same statistical tests revealed that the highest values appeared in the NET region ($p < 0.0001$). As these quantitative observations were consistent with the qualitative results (Figs. 5, 6), it can be concluded that the imaging characteristics associated with the prediction of HGG and LGG are indicated by their localization in the ET and NET regions, respectively. In other words, it is implied that the classification model mainly depends on the number of feature vectors associated with the presence (ET) or absence (NET) of contrast enhancement in the tumor.

## Discussion

Multi-parametric MRI can reveal the morphological heterogeneity of gliomas, which contain various sub-regions (edematous regions, enhancing and non-enhancing tumor cores) with varying histological and genomic phenotypes. This intrinsic heterogeneity can also be observed in imaging phenotypes because their sub-regions exhibit different intensity patterns across different MR sequences. In this study, three different regions were considered. The ET is defined by areas exhibiting hyper-intensity in the Gd-enhanced T1 sequences compared with T1 signals^20^. Such regions generally correspond to areas of contrast enhancement, where contrast leakage caused by blood-brain barrier damage may exist^41,42^. The ED is defined by areas with high T2/FLAIR signal intensity^20^, which represent either low cellularity or edema^43^. The NET indicates non-enhancing tumor regions and pre-necrotic and/or necrotic regions located in the non-enhancing part of the tumor core^20^. The imaging appearance of NET typically exhibits hypo-intensity in the Gd-enhanced T1 sequences compared with T1 signals^20^.

The imaging differences between LGG and HGG have attracted a substantial amount of attention regarding early differential diagnosis. Nevertheless, these differences are still debated. Typically, LGG appears as an area of focal signal abnormality with minimal or no contrast enhancement^44^, and does not cause significant blood–brain barrier disruption, which results in less contrast leakage around the lesions. In contrast, most HGG in Gd-enhanced T1 sequences exhibit moderate to strong contrast enhancement, which reflects the degree of microvascularity and the presence of a disrupted blood–brain barrier^45^. Occasionally, necrosis can be observed inside a tumor, and is an important diagnostic feature for HGG^46^. Furthermore, HGG commonly causes significant damage to the blood-brain barrier, which appears as a large ED area surrounding the tumor core. Therefore, based on the segmentation categories adopted in this study, the presence of NETs in the central region of a tumor surrounded by a small ED region can be considered as a typical LGG characteristic. For HGG, a tumorous lesion represented by ET with or without NET and extensively surrounded by ED areas can be considered as a typical representation.

Based on these considerations, our results are consistent with the known imaging characteristics of LGG and HGG. Particularly, the feature ablation study revealed that NET is the most discriminative component of LGG, whereas ET is the most discriminative component of HGG (Fig. 7). The presence of contrast enhancement (ET) is often considered as a sign of HGG^47^. Therefore, the observation that the classification model captured the presence (ET) or absence (NET) of contrast enhancement in the tumor core is compelling.

Several studies have investigated the classification of glioma grades using deep learning. For example, Yang et al. demonstrated that ImageNet-pretrained deep learning models, such as AlexNet^48^ and GoogleNet^49^, can outperform a comparative model trained from scratch, and achieve a maximum test accuracy above 90%^14^. However, their method requires the manual segmentation of the ROIs before the classification. Recently, Banerjee et al. proposed a deep-learning-based algorithm that incorporates volumetric tumor information and achieves a maximum accuracy of 97%^15^. Similarly, Zhuge et al. proposed a two-step approach to automatically segment brain tumor regions and carry out classification according to the bounded image regions that contain tumors^16^. They also achieved a maximum classification accuracy of 97%. To achieve superior performance, an important aspect of deep-learning-based models is the size and extent of the input images. Banerjee et al.^15^ compared several neural networks using patch-wise, slice-wise, and volume-wise inputs, and achieved glioma grading accuracy of 82%, 86%, and 95%, respectively. Particularly, when considering the input as a 3D volume, these deep-learning-based approaches can outperform machine-learning-based approaches that use logistic regression based on brain tumor radiomics features (accuracy of 88%)^50^.

Compared with previous studies, the classification accuracy of the proposed model is ranked between the accuracy achieved when using slice-wise inputs and the accuracy achieved when using volume-wise inputs^15^. Even though the proposed feature extraction process was performed using slice-wise inputs, the classification model is as simple as using logistic regression. Therefore, the proposed classification model’s performance is remarkable compared with that of end-to-end deep learning models that take slice-wise inputs. Notably, Rudin^51^ insisted that the belief whereby more complex models are more accurate is not always true, particularly when a good representation in terms of meaningful features is constructed for a target task. She also argued that there is often no significant difference between the prediction accuracy achieved by more complex models, such as deep neural networks, and much simpler models, such as logistic regression, when the representative data features are given. Accordingly, we confirmed that the feature vectors obtained from the pre-task of tumor segmentation are sufficiently informative for the discrimination of glioma grading.

To the best of our knowledge, this is the first study that uses vector quantization to obtain a shareable set of feature vectors across a population for the purpose of identifying specific factors associated with clinical information. The reason for acquiring quantized latent representations rather than continuous ones for the deep radiomics is that it can explicitly fix the variability of internal representations of CNNs. As the original radiomics is an approach to extract a large number of quantitative image features for the objective comparison of medical images^8^, we believe it is important to yield a comparable set of latent representations in a dataset even when using deep learning as a feature extraction method. Based on these considerations, our methodology has shown considerable success in extracting deep radiomics from the segmentation model, exploiting them in the glioma grade classification, and visualizing the imaging region encoded by each feature vector significantly attributed to the classification. The observations are consistent with those reported in the literature and can equip physicians with an enhanced understanding of the inner reasoning process of classification models.

### Limitations

This study has several limitations. First, the detailed information on the public dataset, including scanner vendors, the time of scan, field of view, and patient demographic, was unclear. Second, we have not tested the generalizability of the results using external datasets. In order to compensate for these shortcomings, robustness of the proposed method was investigated, and it was shown that the deep radiomics has a certain level of invariance to the shift and scale of the pixel values (Fig. 4). This could be due to the feature normalization being operated in each layer of the segmentation network. Furthermore, the variation of the encoder output caused by the perturbations can also be suppressed by the vector quantization. Third, no direct comparison was conducted with the conventional or advanced techniques using Radiomics^8,52^ and other deep-learning-based feature extraction methods^53^. Moreover, it should be noted that the distinction between LGG and HGG in the BraTS dataset is different from those in the WHO classification of gliomas^2^, as Dequidt et al. clarified^23^. Our source code is publicly available for further research to resolve the aforementioned limitations. Furthermore, future technical challenges include the extension of this work to end-to-end learning including classifiers, and pre-task without label information using self-supervised learning.

## Conclusion

Our deep radiomics approach is a data-driven technique to utilize the internal representations acquired inside deep neural networks as imaging markers for downstream tasks. Vector quantization is the core of our proposal to resolve the internal variability of typical CNNs for extracting a shareable set of feature vectors in a population. Based on the dataset containing brain MRIs with gliomas, we demonstrated that the method could provide a good classification accuracy for the glioma grades as well as interpretability for the task-specific radiological findings on which the classification model depends. The proposed method is versatile and easily applicable to other research fields.

## Data Availability

Data analyzed during the current study are available on Center for Biomedical Image Computing & Analytics (https://www.med.upenn.edu/cbica/).

## Code availability

The source code in this work is publicly available on GitHub (https://github.com/Kaz-K/deep-radiomics-glioma).
